# Alzheimer’s disease as a fundamental disease of information processing systems: An information theory perspective

**DOI:** 10.3389/fnins.2023.1106623

**Published:** 2023-02-10

**Authors:** Myongin Oh, Donald F. Weaver

**Affiliations:** ^1^Krembil Research Institute, University Health Network, Toronto, ON, Canada; ^2^Department of Chemistry, University of Toronto, Toronto, ON, Canada; ^3^Department of Pharmaceutical Sciences, University of Toronto, Toronto, ON, Canada; ^4^Department of Medicine (Neurology), University of Toronto, Toronto, ON, Canada

**Keywords:** Alzheimer’s disease, amyloid-beta, synaptic transmission, cytokine, information theory, psychotherapy

## Abstract

The human brain is a dynamic multiplex of information, both neural (neurotransmitter-to-neuron, involving 1.5×10^15^ action potentials per minute) and immunological (cytokine-to-microglia, providing continuous immune surveillance *via* 1.5×10^10^ immunocompetent cells). This conceptualization highlights the opportunity of exploiting “information” not only in the mechanistic understanding of brain pathology, but also as a potential therapeutic modality. Arising from its parallel yet interconnected proteopathic-immunopathic pathogeneses, Alzheimer’s disease (AD) enables an exploration of the mechanistic and therapeutic contributions of information as a physical process central to brain disease progression. This review first considers the definition of information and its relevance to neurobiology and thermodynamics. Then we focus on the roles of information in AD using its two classical hallmarks. We assess the pathological contributions of β-amyloid peptides to synaptic dysfunction and reconsider this as a source of noise that disrupts information transfer between presynaptic and postsynaptic neurons. Also, we treat the triggers that activate cytokine-microglial brain processes as information-rich three-dimensional patterns, including pathogen-associated molecular patterns and damage-associated molecular patterns. There are structural and functional similarities between neural and immunological information with both fundamentally contributing to brain anatomy and pathology in health and disease. Finally, the role of information as a therapeutic for AD is introduced, particularly cognitive reserve as a prophylactic protective factor and cognitive therapy as a therapeutic contributor to the comprehensive management of ongoing dementia.

## 1. Introduction

There are a number of “duality paradoxes” in the physical sciences, with the wave-particle duality of light being the time-honored example Rab et al. (2017). In neuroscience, the concept of information as a discrete entity offers an analogous duality paradox, being abstract and fundamental, subjective and objective, metaphysical and physical. Considering information as both a non-physical and physical entity within the brain is conceptually complex. The assimilation of information through learning changes the physical structure of the brain, differentially organizing and re-organizing multiple brain regions; conversely, the brain structure dictates our capacity to receive and process information. Neural information is thus not exclusively an abstract entity but rather interactively exists only through its physical representation in the brain, being enabled yet constrained by all the possibilities and restrictions imposed by neuroanatomy; through this representation, information emerges as physical glue at the brain-mind interface. Thus, in the brain, information arguably exists as a tangible physical reality with the ability to influence (and to be influenced by) the brain structure.

The conceptualization of information as a physical entity necessitates its placement, possibly in an overarching position, within the ascending structural hierarchy defined by atoms, molecules, macromolecules, organelles, cells, tissues, and organs. Moreover, this conceptualization highlights the essentially untapped opportunity of exploiting information not only in the mechanistic understanding of disease pathology, but also as a potential therapeutic modality in its own right.

Alzheimer’s disease (AD) is the quintessential neurodegenerative dementia. Arising from its parallel yet interconnected proteopathic-immunopathic pathogeneses (Weaver, 2020), AD prototypically enables an exploration of the mechanistic and therapeutic contributions of information as a physical entity implicated in human disease progression. AD affects the two principal cell lines in the brain: neurons and glia. Extensive neuronal death and brain atrophy (arising in part from pathological protein misfolding) result in impairment of learning, memory, language, perception, and executive function – hallmarks of disordered neural information processing (Guarino et al., 2019). Concomitant microglial activation dysregulates the pro-inflammatory/anti-inflammatory balance culminating in inflammasome-mediated cellular toxicity – a hallmark of disordered immune information processing (Su et al., 2016; Hanslik and Ulland, 2020). AD may therefore be regarded as a chronic, progressive disease characterized by dysfunction in both neural and immunological information systems. This novel and widely encompassing information-based conceptualization of AD not only affords unique perspectives on disease mechanisms, but also underscores the prospect of using information as a therapeutic agent, either prophylactically or acutely, possibly in harmony with conventional pharmacological approaches.

In this article, we present an overview of AD-mediated dysfunction in neural and immunological information systems to provide evidence that AD is an “informational disease (i.e., condition that impairs normal functioning of the brain with information).” In the subsequent sections, we first introduce relevant concepts from information theory and thermodynamics and several applications of information theory in neuroscience including mathematical modeling of neurotransmission. This is then more fully extended to AD, not only from a disease mechanism perspective, but also as a source of insights regarding putative therapeutic approaches.

## 2. Background

Information theory and thermodynamics share entropy as a conceptual pillar. Thermodynamically, a living organism is an open, complex yet self-organizing physical system that displays the characteristics of life by interacting in a temperature-dependent process with its surroundings through a constant exchange of matter, energy, and information while maintaining a dynamic steady state crucial for survival. The state of such a physical system and how it evolves as a function of time and in response to a panoply of external stimuli are prescribed by energetics and combinatorics; ultimately, Nature favors the ability to maintain order, primarily *via* brain-orchestrated processes, to achieve a state of high stability and high probability, aiming for free energy minimization at equilibrium.

In a human brain, biological mechanisms operating at multiple spatial and temporal scales are strictly maintained and manipulated at each hierarchical level of structural organization [including molecules, subcellular organelles, cells, and tissues (Grizzi and Chiriva-Internati, 2005)], forging a massive communication network with no discontinuity. The topology of this network defines the pattern of direction and strength of information flow from one spatiotemporal point to another, either within the system or between the system and its environment, and ensures efficient, reliable communication from information source to destination. Thus, the central nervous system (CNS) enables the brain and mind to communicate actively with the ever-changing outside world while concomitantly maintaining unchanging homeostasis *via* its vast array of neural circuits which input sensory information, process it, and trigger responses. The CNS functions primarily as a collection of channels that convey information as discrete electrical impulses; the convoluted architecture of this channel network is composed of 86 billion neurons and 100 trillion synaptic connections (Korade and Mirnics, 2014), providing the physical basis for the speed and sophistication of information transmission, compression, and processing. Structurally, the intercellular transfer of information involves a cascade of biochemical processes that generate a transmembrane electrochemical gradient and convert the propagation of action potentials (i.e., electrical signals) into the release of neurotransmitters (i.e., chemical signals).

During neurotransmission, synaptic vesicles liberate both neurotransmitters and protons into the synaptic cleft, leading to local, transient extracellular pH fluctuations (Sinning and Hübner, 2013). The movement of neurotransmitters in the brain interstitial fluid is characterized by the intrinsic randomness of Brownian motion (Veletić et al., 2016). Evidence has also accumulated to demonstrate that protons fulfill the criteria as co-transmitters (Du et al., 2014; González-Inchauspe et al., 2017), being involved in regulated intercellular signaling in concert with classical neurotransmission (Soto et al., 2018). Proton translocation in water, the major component of brain interstitial fluid, is likewise described as a random process known as the de Grotthuss (1806) mechanism in which an excess proton hops along a “water wire” (or pre-existing sequence of hydrogen bonds among water molecules) in an exclusive, stepwise manner (Codorniu-Hernández and Kusalik, 2013; Ball, 2017). Hence, the diffusional mode of neurotransmitters and protons in synaptic transmission contributes to the stochastic nature of nerve conduction.

Information transmission in the CNS is thus inherently stochastic, and its likelihood of occurrence, which is fundamentally dependent on the probability of neurotransmitter release with variable timing and amplitude, is a critical factor in the modulation of signal flow in neural networks (Branco and Staras, 2009; Ribrault et al., 2011). In addition to being stochastic, these brain processes are also inherently noisy as evidenced by the fluctuations in repeated measurements of neuronal firing causing neuronal spike trains to be characterized by variability and irregularity; indeed, noise is an inseparable part of experimental brain measurement. This noise arises either from an irreducible indeterminacy or from epistemic limitations, including limitations of measuring known variables and controlling hidden variables (Trappenberg, 2010). Consequently, a probabilistic approach is optimally employed when analyzing and modeling neural information transmission.

Information transmission in the brain is a consequence of the coordinated but probabilistic performance of individual neurons and ensembles thereof; indeed, information is defined in terms of a probability distribution. As atoms are the fundamental building block of matter, information emerges as the fundamental unit of brain function underlying virtually all microscopic and macroscopic neural processes. Damage or “blockage” to this communication network can herald the onset or progression of brain dysfunction.

AD is an irreversible, progressive neurodegenerative disease that arises primarily from communication failure among neurons and among glia. Microscopically, the diseased brain is afflicted at the molecular level with the presence of extracellular amyloid plaques composed of aggregates of Aβ peptides and intraneuronal neurofibrillary tangles of a hyperphosphorylated microtubule-associated tau protein, linked to neuronal death and synaptic loss (Pospich and Raunser, 2017; Chen and Mobley, 2019; DeTure and Dickson, 2019; Lee et al., 2019b). Also, at the cellular level, activated microglia trigger immunopathic responses that contribute to disease progression (Hemonnot et al., 2019; Ennerfelt and Lukens, 2020). Macroscopically, the neuronal and microglial changes are reflected as cerebral atrophy mirroring disease progression (Frisoni et al., 2010; Tondelli et al., 2012; Marino et al., 2019; Frenzel et al., 2020).

The notion that AD is an informational disease is preliminarily supported by brain network analysis and neuroimaging studies. For example, from transgenic mouse experiments, Kashyap et al. (2019) derived a complex network model which suggests that AD progression can be interpreted as a phase transition from initial robustness to irreparable disintegration, and estimated a critical time after which the neuronal network undergoes rapid deterioration. Based on their observation on the loss of spines caused by Aβ accumulation, the model explains that the consequent reduction in synaptic density impairs rapidly coordinated activity of neurons, global efficiency of network signal transmission, and structural plasticity of the network as the disease progresses (Kashyap et al., 2019). Also, Engels et al. (2017) used magnetoencephalography to confirm a posterior-to-anterior information flow over the cortex in higher frequency bands in healthy brains, and found it to be disturbed in both cortical and subcortical regions in early-onset AD brains as highly connected regions (or hubs) in posterior areas are pathologically disrupted. They observed a prominent reduction in the information flow from the precuneus and the visual cortex, toward frontal and subcortical structures, in AD. Thus, abundant existing data provide empirical evidence for AD as an informational disease.

## 3. What is information?

In a seminal paper “*A Mathematical Theory of Communication*” (Shannon, 1948), Claude E. Shannon, the founder of information theory, provides a mathematical definition of information in the context of communication and describes how information can be transmitted between different elements of any system, whether biological or man-made, in an efficient and reliable manner in defiance of noise. Information is what allows one, who is in possession of that information, to make predictions with accuracy better than chance (Adami, 2016) or, simply, reduces uncertainty in a situation where one has to make a choice out of multiple alternatives. The fundamental results from information theory can be summarized as follows: (1) it is impossible to compress data below the entropy bound of the source without losing information; and (2) it is possible to transmit information through a noisy channel at any rate less than channel capacity with an arbitrarily small probability of error (Ash, 2012).

Shannon first introduced the uncertainty function called information entropy *H* which is equal to the weighted average of information contents of all the possible states *i* where the weights are the probabilities *p_i_* of occurrence of the states. The sole function that satisfies the certain characteristics of information is in the following form:


$$H\,\left(p_{1},\cdots ,p_{n}\right)=-k\,\sum_{i}p_{i}\,\ln p_{i}$$

where *k* is a positive constant and the negative logarithm of *p_i_* is the information content associated with the state *i*. The information content can be understood alternatively as the level of surprise when the state is observed. *H*, which is strictly nonzero for discrete random variables, is a measure of the amount of information contained in a probability distribution or reduction in uncertainty when the outcome of a random experiment has been revealed. A bit is thus equal to the amount of information, or the extent of uncertainty, involved in a binary question regarding two equiprobable outcomes as in flipping a fair coin.

The same mathematical expression for entropy occurs both in information theory and statistical mechanics. Are the Shannon entropy and the Boltzmann entropy the same in nature? There is still considerable disagreement over how to relate information to thermodynamic entropy (Ben-Naim, 2015, 2017b; Ben-Naim, 2017a). Since the foundation of the second law of thermodynamics, which states that the entropy of an isolated system never decreases over time, many have strived to incorporate information explicitly into classical thermodynamics and gauge thermodynamic costs for information manipulation. In 1867, James Clerk Maxwell first revealed the relationship between information and entropy in a thought experiment wherein a tiny, intellectual being sorts gas molecules by velocity and thus reverses heat flow by using information about their positions and velocities in two neighboring chambers (Leff and Rex, 2002). At first, Maxwell’s “demon” seems to violate the second law of thermodynamics, but the imaginary creature illustrates that one can utilize information to ease the restrictions imposed by the second law on the exchange of energy between a system and its surroundings (Parrondo et al., 2015).

In the Bayesian paradigm, probability is not an inherent property of a physical system but essentially quantifies the degree of ignorance an observer has about the state of the system as the observer’s estimate of probability is updated whenever new information becomes available (Stone, 2013). Jaynes (1957) reinterpreted statistical mechanics as a form of statistical inference within the framework of subjective probability and demonstrated that the conventional relations, such as the partition function and the free energy, in statistical mechanics are an immediate consequence of the principle of maximum entropy, which is the least biased estimate possible on the given information (Jaynes, 1957). Jaynes derived the equivalence of information entropy to thermodynamic entropy for canonical equilibrium states except for the presence of the Boltzmann constant *k* which may be regarded as a correction factor (Jaynes, 1957).

Landauer speculated that information is explicitly physical and thus obeys the laws of physics since it is stored in physical systems (e.g., brains), transmitted by physical means (e.g., all-or-none action potentials), and processed in physical devices (e.g., neurons) (Landauer, 1991; Lutz and Ciliberto, 2015). Landauer showed that the erasure of information is inexorably accompanied by the generation of heat (Landauer, 2000). Specifically, the erasure of one bit of classical information in a two-state system dissipates a minimum amount of energy proportional to temperature (i.e., *E* = *kTln* 2), known as the Landauer limit, as heat to compensate the entropic loss (Plenio and Vitelli, 2001; Roy, 2014). The physical nature of information ensures that it can be included in the second law of thermodynamics not as a pure abstraction, and by extension, information processes (e.g., erasure and measurement) can be treated as physical operations with thermodynamic costs (Parrondo et al., 2015).

It was also suggested that the second law of thermodynamics operates at the level of information; that is, information is erased by some processes and cannot be recovered once erased, and the dynamics of information is related to but independent of the dynamics of energy (Duncan and Semura, 2004). The applicability of the first statement to AD is questionable since it has been reported that memory failure in early AD models reflects an impairment in the retrieval of information rather than the erasure of information (Roy et al., 2016). Based on the central ideas that (1) information is a fundamental physical quantity, and (2) temperature connects information and energy, heat transfer can be viewed as a loss of detailed information about the state of a system, and hence, there is a direct link between heat flow Δ*Q* and information loss Δ*I* (Duncan and Semura, 2004):


$$\Delta \,I=-\frac{\Delta \,Q}{k\,T\,\ln2}.$$

Based on the fact that information often drives physical systems away from equilibrium, thermodynamics of information can be translated in terms of non-equilibrium thermodynamics (Parrondo et al., 2015). Stochastic thermodynamics rigorously show that Shannon entropy determines the energetics of a non-equilibrium process coupled to thermal reservoirs of constant temperature *T* (Seifert, 2012). For example, when an observer acquires new information about a physical system with states *x* after measurement, the statistical state shifts from *p*(*x*) to *p*(*x*|*m*) where *m* is the measurement outcome. The post-measurement state is generally out of equilibrium even if the pre-measurement state was in equilibrium. Assuming that measurement does not affect the Hamiltonian and the state of the system, the increase in non-equilibrium free energy *F* is given by Lloyd (1989), Parrondo et al. (2015).


$$\Delta \,F_{m}=-T\,\Delta \,S_{m}=k\,T\,I\,\left(X;M\right)>0$$

where *I*(*X*;*M*) is the mutual information between the state *X* and the measurement outcome *M*. Since the mutual information is positive, information acquisition (i.e., measurement) always increases the free energy and thus the amount of extractable work in an isothermal process (Parrondo et al., 2015). Detailed discussions of the connection between information and thermodynamics and the realization of the physical nature of information have been presented in other comprehensive reviews (Serreli et al., 2007; Maruyama et al., 2009; Raizen, 2009; Toyabe et al., 2010; Bérut et al., 2012; Lutz and Ciliberto, 2015; Parrondo et al., 2015; Rex, 2017).

## 4. Information theory and neuroscience

After Shannon’s (1948) seminal work, the flexibility of information theory enabled its applicability to a diversity of research areas outside its original scope (even though Shannon (1956) alerted against the “injudicious” use of information as a novel tool in his essay *The Bandwagon*). Accordingly, information theory has been adopted in neuroscience as a primary means to quantify neural information and to evaluate the performance of neurons and their circuits. The first application was made in MacKay and McCulloch (1952) who analyzed neural coding from the perspective of information theory to estimate the upper bounds on the information transmission capacity (i.e., channel capacity) of a neuron assuming two types of coding, namely, pulse code modulation and pulse interval modulation. They found that a system of the latter type can signal several times more information per second through synaptic transmission than the former type.

Assuming a neuron is a communication channel, neural coding is concerned with measuring how much information neural spikes carry about the stimuli that evoke them and characterizing their relationship (i.e., stimulus-response models) (Borst and Theunissen, 1999). Neural coding capacity is the maximum output entropy rate possible at the mean spike rate (MacKay and McCulloch, 1952; Koch et al., 2004; Koch et al., 2006). The actual capacity that is related to a neuron’s inputs is smaller than the coding capacity because [1] the output entropy includes noise entropy, and [2] the consecutive spikes are not always mutually independent (Koch et al., 2004; Stone, 2018). Neural coding efficiency is a measure of the proportion of entropy in a neuron’s output that comprises information about its inputs (Rieke and Warland, 1999; Stone, 2018).

A compelling attempt has been made to discern the existence of theoretical connections between thermodynamics and information theory permitting the unification of brain and cognition models. Thermodynamic terms have been frequently used to model brain activity (Salerian, 2010; Varpula et al., 2013), while information-based approaches have been developed to describe cognitive processes (Friston, 2010; de Castro, 2013). Two important features are commonly involved in thermodynamic models of brain activity (Collell and Fauquet, 2015): first, the second law of thermodynamics is the main principle that drives neural activity; second, the brain is a dissipative structure in which an entropic exchange takes place across its boundaries, and a source of free energy (mainly in the form of adenosine triphosphate, ATP) is required to maintain a reproducible steady state (e.g., to transmit a new train of neural spikes). The core principle that lies in the information-based model proposed by Friston et al. (2006), Friston (2010) is the so-called free energy principle which asserts that any self-organizing system that is at equilibrium with its environment must minimize its free energy. In his theory, free energy is defined as the upper bound on entropy or “surprise” associated with receiving a sensory input and having a model of the world (Collell and Fauquet, 2015). Self-organizing biological agents, including brains, should restrict themselves to a limited number of states by averting surprising states (i.e., minimize the long-term average of surprise) to ensure that their sensory entropy remains low (Friston, 2010). Collell and Fauquet (2015) subsequently proposed a theoretical framework to formalize the connection between neural activity and cognition by means of the classical links between thermodynamics and information theory. A comprehensive review on thermodynamic relationships in the brain has been presented by Street (2016).

## 5. Information transmission in the brain

### 5.1. Neural information

Functionally, the brain is a complex system in which neural information is disseminated and distributed *via* interneuronal communication processes. Traditionally, such communication processes have been represented by a simple linear model known as the Shannon–Weaver model of communication (Shannon et al., 1949), which may be deconstructed into six key components. The sender is an information source that generates a message to be communicated. The encoder associates with each message an object which is suitable for transmission over a channel and less susceptible to channel noise. The encoded message is then transmitted over the channel. The decoder operates on the channel output to recover the original message that is acceptable to the receiver. When the sender and the receiver communicate each other through a noisy channel, the recovery of the original message with complete reliability is normally unattainable by virtue of the influence of noise (Ash, 2012), which is a general term for anything that is prone to disturb signals and thus produce errors in the course of transmission. The performance of each component in the communication model, and thus information transmission, cannot be deterministic but must be statistically defined as clearly seen in the definition of information entropy (Reza, 1994).

**FIGURE 1:**
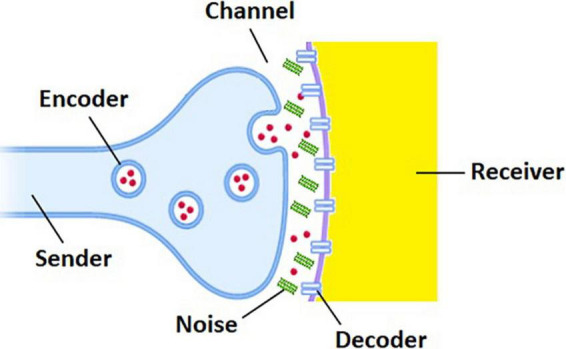
Shannon–Weaver model of communication for neurotransmission disrupted by the presence of Aβ oligomers (green bars) in the synaptic cleft between presynaptic (blue) and postsynaptic (yellow) neurons. Red circles and blue bars represent neurotransmitters and receptors, respectively.

The beauty of the Shannon–Weaver model is found in its generality that encompasses all communication processes regardless of whether signals are digital or analog or whether the communication system is artificial or biological. Since neurotransmission is probabilistic and noisy (Smetters and Zador, 1996; Branco and Staras, 2009; Yarom and Hounsgaard, 2011; Veletić et al., 2016), information transfer between synaptically coupled neurons can be formulated in terms of the Shannon–Weaver model as follows (Figure 1): (1) the presynaptic and postsynaptic neurons are the sender and the receiver, respectively, conveying action potentials as discrete messages. (2) Neurotransmitter-generating transporters or molecular mechanisms that create synaptic vesicles and neurotransmitters serve as the encoder. (3) The channel is the extracellular aqueous medium in the synaptic cleft. (4) The receptors on the postsynaptic neuron or their ligand-binding mechanisms function as the decoder. (5) The sources of noise in the nervous system arise from the molecular to the behavioral level (Faisal et al., 2008). For example, the presence of Aβ oligomers in the synaptic cleft can be a fatal source of noise in AD brains (discussed below).

Veletić et al. (2016) applied information theory to model neurotransmission at a single synapse and estimated the synaptic channel capacity. In their model, both spike and neurotransmitter sequences are described as non-homogeneous Poisson processes. They identified three distinct sources of unreliability in the neurotransmitter release machinery: (1) the release of neurotransmitters upon the arrival of an action potential, which is modulated by the vesicle release probability, driven by the intracellular Ca^2+^ concentration within the presynaptic terminal, (2) the propagation of neurotransmitters toward the postsynaptic receptors, which is described by the neurotransmitter propagation probability following a Bernoulli distribution, and (3) the binding of neurotransmitters to the receptors whose probability can also be modeled to follow a Bernoulli distribution in a simplified scenario. They computed the channel capacity of noisy Poisson-type bipartite and tripartite synapses with varying conditions of vesicle releases, through the analogy between optical and synaptic communication systems. The detailed calculations are presented in the work of Veletić et al. (2016), and the extension of their work to neurotransmission over multiple-access synaptic channels (consisting of multiple synapses that link two neurons and operate jointly) is found in the work of Veletić and Balasingham (2020). Other theoretical works that involve rigorous information-theoretical analysis on synaptic transmission include a derivation of lower bounds on the capacity of a simple model of a cortical synapse (Manwani and Koch, 2001), estimation of lower and upper bounds on the rate of information transmission in a model of synaptic facilitation (Salmasi et al., 2019), and development of a realistic model predicting the dynamics of neurotransmission at the synapse between the mossy fiber and the granule cell in the cerebellum (D’Angelo et al., 2005).

### 5.2. Immunological information

In the immune system, information exists in spatiotemporal patterns. The recognition, learning, storage, communication, and transformation of these patterns ultimately shape and control the behavior of the immune system and how it responds to a diversity of injurious threats. Multiple informational design principles are present in the brain’s immune system, characterized by being diverse, distributed, dynamic, adaptable, error tolerant, and putatively self-protective.

Structurally, the neuroimmune system comprises microglia as its cellular backbone and cytokines as its molecular backbone. Cytokines are a group of diverse small proteins (4–20 kDa) which function as immunomodulatory signaling molecules regulating immunity by inducing changes in gene expression and by influencing the responsiveness of selected cell populations (Kany et al., 2019). Most cytokines enhance or inhibit the action of other cytokines through a complex interdependency that involves pleiotropism, redundancy, and synergism (de Haan et al., 1996).

Somewhat analogous to neurotransmitters, cytokines are released and diffuse to receptor proteins that may be located on the originating host microglial cell or on adjacent neuroglial cells. The same cytokine may induce different effects on different microglial cells; conversely, different cytokines may elicit similar biological responses. The probabilistic release and binding of cytokines demonstrate stochastic and noisy behavior of the immunological information. The triggers that activate cytokine-microglial brain processes are information-rich three-dimensional patterns, including pathogen-associated molecular patterns (PAMPs) and damage-associated molecular patterns (DAMPs) (Venegas and Heneka, 2017). PAMPs are “non-self” molecular motifs (e.g., glycans) found within microbes that are recognized by pattern recognition receptors (PRRs) in immune cells, heralding a microglial response. DAMPs are “self” molecular motifs (e.g., nuclear or cytosolic proteins from injured cells) that are also recognized by PRRs, thereby perpetuating a non-infectious inflammatory response. Thus, at a high-level generalization, there are similarities between the structural and functional underpinnings of neural and immunological information with both fundamentally contributing to brain anatomy and pathology in health and disease.

## 6. Information in the pathogenesis of Alzheimer’s disease

### 6.1. Neural information

The connection between two neurons is characterized by synaptic multiplicity and variability. It is composed of multiple synaptic contacts which can be functionally heterogeneous even when they belong to the same presynaptic axon and target the same postsynaptic neuron (Branco and Staras, 2009). The strength of a neuronal connection rests upon three main factors: the number of synaptic contacts, the magnitude of the postsynaptic depolarization caused by neurotransmitters liberated from a single synaptic vesicle, and the likelihood of neurotransmitter release at each synapse (del Castillo and Katz, 1954). Experimental evidence shows that multiple synapses contributing to a single connection can exhibit a broad and continuous probability distribution of neurotransmitter release (Murthy et al., 1997); this probability is so dynamic it can change over a short timescale (Zucker and Regehr, 2002). Furthermore, the probability is regulated with high spatial precision, and its tuning is the result of a complex series of molecular and cellular processes (Branco and Staras, 2009).

The accumulation of amyloid plaques and neurofibrillary tangles is a classical phenotypic hallmark of AD, traditionally classified as a protein-misfolding disease, or proteopathy, since the toxic deposits are composed of misfolded protein aggregates, which can be seeded *via* a prion-like mechanism (Pospich and Raunser, 2017). Soluble Aβ oligomers, which build a complex equilibrium with insoluble Aβ fibrils, are key neurotoxins in AD brains. Multiple lines of evidence show that Aβ peptides exert an adverse impact on multiple cellular and subcellular brain processes; for example, an imbalance between the production and clearance of Aβ peptides, particularly those that are highly prone to oligomerization, precedes abnormal synaptic pruning and gliosis marked by increases in activated microglia (microgliosis) and reactive astrocytes (astrocytosis) (Frost and Li, 2017; Olsen et al., 2018; Selkoe, 2019). Aβ peptides also trigger calcium dyshomeostasis and oxidative stress by enhancing free radical generation (Arbel-Ornath et al., 2017; Butterfield and Boyd-Kimball, 2018; Marsh and Alifragis, 2018). Moreover, Aβ-induced actin cytoskeletal abnormalities (Lee et al., 2019a) and mitochondrial dysfunction (Correia et al., 2016) have been reported, and it was found that proteasome dysfunction correlates with the detection of intraneuronal Aβ oligomers (Tseng et al., 2008). Also, extensive evidence supports the pathological role of Aβ peptides in synaptic dysfunction (Shankar and Walsh, 2009; Forner et al., 2017; Marsh and Alifragis, 2018; Fagiani et al., 2019). Collectively, these processes implicate Aβ as a causative factor of interneuronal information communication failure in AD, particularly by defects in synaptic vesicle dynamics and neurotransmitter action. It is, however, well known that the presence of Aβ alone is not informative as to where patients stand along a putative pathway of preclinical AD progression; those with evidence of both Aβ and biomarkers suggestive of neurodegeneration seems to show the greatest risk of cognitive decline (Jagust, 2015).

When soluble Aβ oligomers interfere with the reuptake of extracellular glutamate, they undermine synaptic function through hyperexcitability of glutamatergic neurons as evidenced by the occurrence of seizures in AD patients (Vossel et al., 2013; Lam et al., 2017) and neuronal hyperactivation in the neocortex and hippocampus where Aβ accumulates in abundance (Zott et al., 2018). Zott et al. (2019) used mouse models of AD to demonstrate that Aβ-mediated hyperactivation is initiated by the suppression of glutamate reuptake by neurons and astrocytes, which is linked to a defect in synaptic transmission exclusively in active neurons. They also reported that the infusion of human Aβ oligomers into the hippocampus results in hyperactivation in a mechanistically similar fashion to a glutamate reuptake blocker (TBOA). An excessive amount of the excitatory neurotransmitter then triggers excitotoxicity and eventually the degeneration of dendrites and cell death (Mattson, 2019).

Aβ-mediated hyperactivation is a typical example that illustrates the perturbing effect of Aβ oligomers on information transmission at synapses in the affected brain. It can be thought of as the noise (i.e., Aβ-induced glutamate reuptake inhibition) affecting directly on the performance of the decoder (e.g., AMPA and NMDA receptors). From the perspective of information theory, it is also interesting to understand how Aβ oligomers would alter the probability of neurotransmitter release, one of the critical sources of the stochasticity of neurotransmission, which is intimately related to the performance of the encoder.

Aberrant neurotransmitter release induced by Aβ oligomers has been extensively reported. Recently, He et al. (2019) found that a significant reduction in the probability of neurotransmitter release at the hippocampal synapse between Schaffer collateral and CA1 pyramidal neurons in mouse models of AD with elevated Aβ production leading to an mGluR5-mediated presynaptic depletion of phosphatidylinositol-4,5-bisphosphate in axons. The same observation was made when synthetic Aβ oligomers were present at the synapse in wild-type mice (He et al., 2019).

Several studies report that Aβ peptides affect the activity of key proteins involved in either the signaling mechanism that regulates the availability and recovery of synaptic vesicles in neurotransmitter release or the interaction between synaptic vesicles and the presynaptic membrane (Marsh and Alifragis, 2018). For example, Park et al. (2017) provided evidence that the exposure of neurons to soluble Aβ hampers trafficking and reallocation of synaptic vesicles among synapses *via* activation of calcium/calmodulin-dependent protein kinase type IV (CaMKIV), thereby preventing neurons from physiological synaptogenesis and synaptic plasticity. Using *in vitro* binding assays and *in vitro* single-vesicle content-mixing assays, Yang et al. (2015) showed that intracellular Aβ oligomers impair the formation of the SNARE (soluble N-ethylmaleimide-sensitive factor attachment protein receptor) complex and thus inhibit SNARE-mediated exocytosis, which is essential for synaptic transmission, by directly binding to the SNARE motif of syntaxin 1a.

Synaptic connections may also play a role in “transmissible” Aβ aggregation within the diseased brain. Premature formation of amyloid plaques can be initiated by the intracerebral infusion of Aβ-rich brain extracts (Jucker and Walker, 2013). The proteinaceous seeds of Aβ aggregation in one region then travel along the axon and propagate to axonally coupled neurons, resulting in the spread of Aβ aggregation to other regions, including neocortical and subcortical regions, similar to those affected in AD (Hamaguchi et al., 2012; Jucker and Walker, 2013; Spires-Jones and Hyman, 2014). The Aβ seeds can therefore serve as self-propagating agents for the actuation and progression of the disease.

Sensory perception is the ability of an organism to detect, process, and respond to internal and external stimuli using traditional (sight, smell, hearing, taste, and touch) and other senses (thermoception, proprioception, nociception, equilibrioception, and mechanoception). Sensory activation transforms physicochemical stimuli into action potentials (sensory transduction) by sensory receptors in the central nervous system. Significant alterations in sensory perception may arise from pathological changes in AD brains. Particularly, there is emerging evidence that olfactory dysfunction is associated with cognitive decline and neurodegeneration in the brain. The sense of smell has shown the greatest promise among all sensory biomarkers of AD since Esiri and Wilcock (1984) observed collections of neurofibrillary tangles in the anterior olfactory nucleus of AD patients (Romano et al., 2021). For instance, odor identification, odor familiarity, and odor recognition memory have been shown to discriminate between cognitively normal individuals, mild cognitive impairment (MCI) patients, AD patients, and those at risk for AD (Olofsson et al., 2010; Albers et al., 2015; Roberts et al., 2016; Yaffe et al., 2017; Dintica et al., 2019; Murphy, 2019). Detailed information is available in many comprehensive reviews including Romano et al. (2021) and Murphy (2019). However, to our best knowledge, it still remains elusive at the cellular and subcellular levels why olfaction deteriorates more significantly compared to other senses [i.e., possible mechanisms that underlie olfactory dysfunction in association with cognitive impairment, AD dementia, and its pathologies (Dintica et al., 2019)] and thus how information theory can be applied to pathological mechanisms that lead to olfactory dysfunction in AD. Also, in the narrative review published in Romano et al. (2021), the authors report that “*only olfaction has been studied to any extent, leaving a clear gap in the literature for the use of other senses.*” Nevertheless, information and coding theory-based approaches have been attempted to understand olfaction at neuronal resolution. The olfactory system encodes and translates information from the vast order space into an accurate neural map in the brain (Grabe and Sachse, 2018). To explore how olfactory signals (e.g., the type and concentration of odorants) are encoded, transformed, integrated, and conveyed at the level of the primary neurons, the notion of olfactory coding has been introduced and widely employed (Ressler et al., 1994; Malnic et al., 1999; Seki et al., 2017; Grabe and Sachse, 2018). The olfactory code contains spatial and temporal dimensions (Giurfa, 2009; Whalley, 2013), and different olfactory coding schemes have been investigated (Schild, 1988; Malnic et al., 1999; Al Yamani et al., 2012; Wilson et al., 2017). But the first application of concepts of information and coding theory to the olfactory system dates back to 1954. Hainer et al. (1954) constructed a theory of olfaction that considers the informational aspects of three domains of knowledge (the subjective olfactory experience, the neurophysiology of the olfactory system, and the requirements for the storage and transmission of information) with many simplifications and specific assumptions concerning only single species (Hainer et al., 1954; Schild, 1988). To understand the significance of the action of odorant molecules on the receptors, they evaluate the information channel capacity of the nerves which connect the olfactory patch (receptor cells) and the cerebrum (mitral cells) and compare it for different patterns with the subjectively perceived and counted information of olfaction (i.e., the number of odors and intensity differences) (Hainer et al., 1954).

A cholinergic hypothesis posits the degeneration of cholinergic neurons in the basal forebrain and the loss of cholinergic transmission in the cerebral cortex and other regions as the major correlate of cognitive dysfunction in AD patients (Kar et al., 2004). A disturbing, suppressive effect of Aβ peptides on acetylcholine synthesis and release was observed (Pedersen et al., 1996; Majdi et al., 2020). It was also found that Aβ peptides inhibit vesicular acetylcholine transporter, thereby preventing its axonal transportation, and reduce choline reuptake (Majdi et al., 2020). Cholinesterase inhibitors (such as donepezil, galantamine, rivastigmine, and tacrine) are known to partially improve cognitive symptoms as they increase acetylcholine levels in the synaptic cleft and enhance cholinergic transmission directly by inhibiting the acetylcholinesterase, an enzyme that hydrolyzes acetylcholine, and thus slowing down the metabolic breakdown of acetylcholine (Raina et al., 2008; Sun et al., 2008; Anand and Singh, 2013; Ferreira-Vieira et al., 2016). Interestingly, the action of the inhibitors is analogous to how an error correction code works in coding theory, which is an encoding scheme that transmits messages (acetylcholine neurotransmission) such that errors (Aβ-induced reduction in acetylcholine production) can be detected and corrected (inhibition of acetylcholine breakdown) within certain limitations [positive effects of the drugs only for a short period of time (Sun et al., 2008; Ferreira-Vieira et al., 2016)] to recover the original message (increase in acetylcholine levels).

### 6.2. Immunological information

Immunological information encoded in various forms may constitute a risk for AD and may initiate the AD cascade, invoking neuroinflammatory processes. The brain responds to a wide range of different immunomodulatory stimuli. Many stimuli – infection, trauma, ischemia, pollution, depression, alcohol abuse – are potentially noxious to the brain (and are known risk factors for AD), triggering an immune response which includes the release of Aβ. Though quite different, these diverse noxious stimuli can be generalized in information terms as being pathogen-/damage-associated molecular pattern (PAMP/DAMP) information stimuli. Significantly, these stimuli need not be applied directly to the brain; for example, multiple animal studies have shown that bacterial lipopolysaccharides (LPS) given peripherally can centrally affect the brain (Banks and Robinson, 2010). However, stimuli that are in the brain [e.g., stroke and head trauma (Jassam et al., 2017)], or anatomically close to the brain [e.g., *Porphyromonas gingivalis* bacteria in the nasopharyngeal-olfactory cavity (Olsen et al., 2016)] may be more potent displayers of PAMP/DAMP informational motifs.

Once a risk factor information trigger aberrantly activates the innate immune system within the brain, additional immunological information errors join the process to enable disease elaboration. Normally, there is a homeostatic balance between pro-inflammatory and anti-inflammatory cytokines within the brain leading to a balanced overall inflammation information state. However, in AD, this balance is skewed leading to excessive pro-inflammatory cytokine messages and culminating in cytotoxic immunotoxicity (Su et al., 2016).

## 7. Information as a therapeutic for Alzheimer’s disease

This realization, that AD can be formalized as an informational disease construct, emphasizes the need to incorporate non-pharmacological approaches when designing therapeutic strategies for AD. Traditionally, therapeutic tactics have pursued a “fight fire with fire” mindset; accordingly, an informational disease should be treated with “information” as a therapeutic modality. Using information as a therapeutic is not without precedent but does require broadening conventional attitudes, particularly within the context of neurotherapeutics for AD.

The brain is the most complex human organ, and dementia is one of the most complex diseases of this organ. From a comparative perspective, few would argue that arterial hypertension is mechanistically much simpler than AD; and yet unlike the situation with AD, no one expects a single “magic bullet” pill for hypertension. Elevated blood pressure is managed comprehensively by risk reduction, lifestyle modification, and often through the use of multiple complementary drugs targeting different receptors in the mechanistic cascade of hypertension. An analogous integrated approach needs to be practiced for AD. Information can be incorporated into this multi-faceted therapeutic stratagem for AD in multiple ways.

The concept of “therapeutic information” can be employed from both a prophylactic risk reduction perspective and as a treatment for active on-going disease. For risk reduction, both neural and immunological information manipulation can be exploited. Cognitive reserve is the hypothesis describing the mind’s resistance and resilience to damage of the brain [the mind is a complex function, an algebraic sum of many functions of the brain (Hansotia, 2003)]. Cognitive reserve indicates the adaptability of cognitive processes that helps to explain differential susceptibility of individual’s cognitive abilities to cope better with brain pathology (Stern et al., 2020). The term endeavors to account for the observation that during later life, those higher in experiential resources [e.g., information acquisition through education and information storage as knowledge (Ruthirakuhan et al., 2012)] enjoy neuroprotective benefits and reduced cognitive decline in aging and disease (Stern, 2012). As a possible mechanism for cognitive reserve, neural reserve theory posits an interindividual variability in brain networks that serve as a basis of any task (Stern, 2009, 2012; Šneidere et al., 2020). An individual engages neural reserve in completion of a specifically challenging task such that neural activity would work more efficiently and thus consume less energy (Šneidere et al., 2020). For an individual who suffers from brain pathology (e.g., AD and traumatic brain injury), alternate brain structures or network (which are unrelated but relatively intact) can replace for the specific performance (Šneidere et al., 2020). Interestingly, it was reported that the density of noradrenergic neurons in the locus ceruleus may be a structural component of neural reserve (Wilson et al., 2013). Although the precise type of information required for an optimal cognitive reserve benefit remains incompletely elucidated, multilingualism has been suggested as a protective factor against dementia and AD (Duncan et al., 2018). It is intriguing that Kim et al. (2019) suggested potential neurological mechanisms by which bilingualism delays cognitive decline associated with AD based on evidence of clinical and structural changes: enhancement of neurogenesis, synaptogenesis, and functional connectivity and increases in white matter integrity and gray matter density. In terms of immunological information, identifying information triggers that herald the initiation of the AD process would be of value in devising public health policy to reduce AD prevalence. As an example, it has been recently suggested that air and noise pollution can be risk factors for AD and dementia (Carey et al., 2018; Younan et al., 2019). Long-term exposure to airborne pollutants (nitrogen dioxide and particulate matter) was associated with higher levels of brain Aβ deposition and cerebrospinal fluid (CSF) neurofilament light protein in a population of cognitively unimpaired adults with increased risk of AD (Alemany et al., 2021) and in older adults with cognitive impairment (Iaccarino et al., 2021). Moreover, animal experiments have associated chronic noise exposure with tau hyperphosphorylation and AD-like pathological changes. For instance, it was reported that noise pollution can induce hyperphosphorylated tau and formation of its pathological neurofibrillary tangle in the rat hippocampus and prefrontal cortex and impair the learning and memory ability of mice (Cheng et al., 2011; Cui et al., 2012). The impact of noise on the immune system is comprehensively reviewed in Zhang et al. (2021). The brain’s immune response to the molecular pattern information triggered by pollutant exposure indicates the value of recognizing and avoiding dangerous environmental triggers. All these observations support the effectiveness of therapeutic information in preventing, minimizing, or delaying the chances of neurodegeneration-induced loss of information and occlusion of information flow in the brain.

As a treatment for active on-going disease, information manipulation may also be a consideration. Multiple forms of cognitive therapy and cognitive behavioral therapy (CBT) have produced mixed results (Carrion et al., 2018), and, although a definitive conclusion on their utility has yet to be reached, they have potential worthy of additional study: [1] Cognition-oriented treatments (e.g., Cognitive Retention Therapy or the Ashby Memory Method) attempt to restore cognitive deficits by reality orientation (*via* presentation of information about person, place, or time) and cognitive retraining (*via* presentation of information with which to exercise mental abilities). [2] Stimulation-oriented treatments [e.g., Cognitive Stimulation Therapy (Rai et al., 2018)] attempt to improve mood, behavior, and function through information-enriched recreational activities, exercise, music, art, and pet therapies. Information manipulation within an AD-afflicted brain may also be achieved by surgical approaches [e.g., deep brain stimulation of the fornix to ameliorate cognitive symptoms (Hescham et al., 2017), or vagus nerve stimulation to improve both cognition and microglial function (Sjogren et al., 2002; Kaczmarczyk et al., 2018)] or pharmacologically [e.g., administration of the anti-epileptic drug levetiracetam to improve cognition and to reduce neuronal overexcitation mediated by excitatory neurotransmitters (Sanchez et al., 2012; Sanz-Blasco et al., 2016)].

A rational multimodal approach to AD should conceivably include therapies at multiple structural levels within the disease mechanism. Consequently, adding information-based therapies to conventional pharmacological approaches would be rational polytherapy (akin to risk management, lifestyle modification, and pharmaceutics in the treatment of hypertension).

## 8. Discussion

Neurons communicate with one another across synapses in a probabilistic manner *via* interconversion between electrical messages (i.e., action potentials) and chemical signals (i.e., neurotransmitters). The nervous system is an example of discrete communication over a noisy channel. The stochastic nature of the reactive and diffusive processes involved in neurotransmission characterizes synapses as an unreliable and noisy channel whose information capacity may be vulnerable to other sources of noise. Mounting evidence indicates that in AD synaptic transmission is prone to errors in the presence of soluble Aβ oligomers, which can interfere with the encoding process in the presynaptic neuron (e.g., synaptic vesicle dynamics and probability of neurotransmitter release) and the decoding process in the postsynaptic neuron (e.g., hyperactivation). Aβ-induced synaptic dysfunction is a causative factor of communication failure among neurons in AD, and this contributes to the notion of AD as an informational disease in the sense that the original message cannot be transmitted intact from one neuron to the other.

Starting from the definition of information as a physical entity, this article also reviewed some significant examples of the theoretical attempts to formalize the relationship between information and energy in the context of neurobiology for a unified theory of the physical brain, and to build mathematical models for information transfer at synapses *via* neurotransmission. Can we integrate Aβ-mediated defects in synaptic transmission as a set of parameters into their models? To answer this question, we believe that a detailed comprehension of the physiological roles of Aβ peptides in the brain and their pathological contributions to the progression of AD is required at both microscopic and macroscopic scales. In addition, we hope that rethinking AD as an informational disease may give a useful insight on potential therapeutic targets and strategies.

Cytokines in the immune system are analogous to neurotransmitters in the neural system. There are structural and functional similarities between neural and immunological information with both fundamentally contributing to brain anatomy and pathology in health and disease. Many risk factors for AD trigger an immune response, including the release of Aβ, and they can be generalized in information terms as being PAMP/DAMP information stimuli. These stimuli need not be applied directly to the brain, and there is a homeostatic imbalance which leads to excessive pro-inflammatory cytokine messages and culminating in cytotoxic immunotoxicity in AD.

The success of unification of all the major hallmarks of AD within the proposed framework is completely determined by the level of our current understanding of relevant neuropathological mechanisms as well as a proper selection of the communication system (or process) to model. For example, mutual information has been used to evaluate functional brain connectivity and quantify the probability of information transmission over brain connections between different cortical regions (Wang et al., 2015; Sayood, 2018; Si et al., 2019). How information theory has been used to study cognition over the last seven decades is well described in the comprehensive reviews by Sayood (2018) and Crupi et al. (2018). Also, it was reported that fatty acids can regulate the number of receptors on microglial cells and thus the inflammatory stage of microglia (Desale and Chinnathambi, 2020). In the simplest form, the switching of microglial phenotypes can be modeled with a binary asymmetric channel with M1 (classical) and M2 (alternative) phenotypes as inputs and outputs and fatty acids as error sources. In a similar fashion, we believe that information theoretical approaches can be constructed to model within the proposed framework other significant hallmarks of AD, including accumulation of tangles of abnormally hyperphosphorylated tau (Mazanetz and Fischer, 2007), degeneration of noradrenergic neurons of the locus coeruleus (Chen et al., 2022) and cholinergic neurons in the basal forebrain (Auld et al., 2002), and development of insulin resistance (Ferreira et al., 2018).

We need to go beyond the perhaps naïve expectation that a single magic-bullet drug is an attainable goal for AD. Accepting the inherent complexity of brain and brain disease, especially dementia, demands that we embrace a “full court press” when tackling AD, at all levels of the structural hierarchy, including therapeutic information, and that we adopt a multi-modal strategy when implementing these informational approaches.

## Author contributions

MO and DW wrote and revised the manuscript. Both authors contributed to the article and approved the submitted version.
